# Reductive Defluorination and Mechanochemical Decomposition of Per- and Polyfluoroalkyl Substances (PFASs): From Present Knowledge to Future Remediation Concepts

**DOI:** 10.3390/ijerph17197242

**Published:** 2020-10-03

**Authors:** Philipp Roesch, Christian Vogel, Franz-Georg Simon

**Affiliations:** Bundesanstalt für Materialforschung und -prüfung (BAM), Division 4.3 Contaminant Transfer and Environmental Technologies, Unter den Eichen 87, 12205 Berlin, Germany; christian.vogel@bam.de

**Keywords:** PFAS, reductive defluorination, reductive decomposition, mechanochemistry, remediation, review, mechanism, fluoride, persistent organic pollutant (POP)

## Abstract

Over the past two decades, per- and polyfluoroalkyl substances (PFASs) have emerged as worldwide environmental contaminants, calling out for sophisticated treatment, decomposition and remediation strategies. In order to mineralize PFAS pollutants, the incineration of contaminated material is a state-of-the-art process, but more cost-effective and sustainable technologies are inevitable for the future. Within this review, various methods for the reductive defluorination of PFASs were inspected. In addition to this, the role of mechanochemistry is highlighted with regard to its major potential in reductive defluorination reactions and degradation of pollutants. In order to get a comprehensive understanding of the involved reactions, their mechanistic pathways are pointed out. Comparisons between existing PFAS decomposition reactions and reductive approaches are discussed in detail, regarding their applicability in possible remediation processes. This article provides a solid overview of the most recent research methods and offers guidelines for future research directions.

## 1. Introduction

Per- and polyfluoroalkylated substances (short PFASs), especially fluorinated organic surfactants, have emerged as a controversial compound class in the past decades. Anthropologically developed in the late 1940s for non-commercial use, PFASs were gradually established as versatile coatings of food-packings [1,2], outdoor apparel [3], as additives in chemical production (such as Teflon® production) [4,5] or highly effective flame retardants. The latter are often referred to as aqueous film-forming foams (AFFFs), since fluorinated telomers, alkyl sulfonates as well as alkyl acids are applied as foam fire extinguishing agents against liquid fires (“Class B” fires) [6]. Due to their unique and remarkable properties, the majority of applied PFASs seem to be irreplaceable [7,8]. The increasing use of PFASs in day to day applications evidently lead to their increased environmental exposure, causing the substance class of PFASs to be under special observation over the last decade. Since long-chained perfluoro alkyl acids, sulfonic acids (C_8_-C_14_) as well as fluorinated alkyl ammonium and phosphates are amongst the main responsible environmental pollutants, research investigations were mainly focused on them [6].

**Figure 1:**
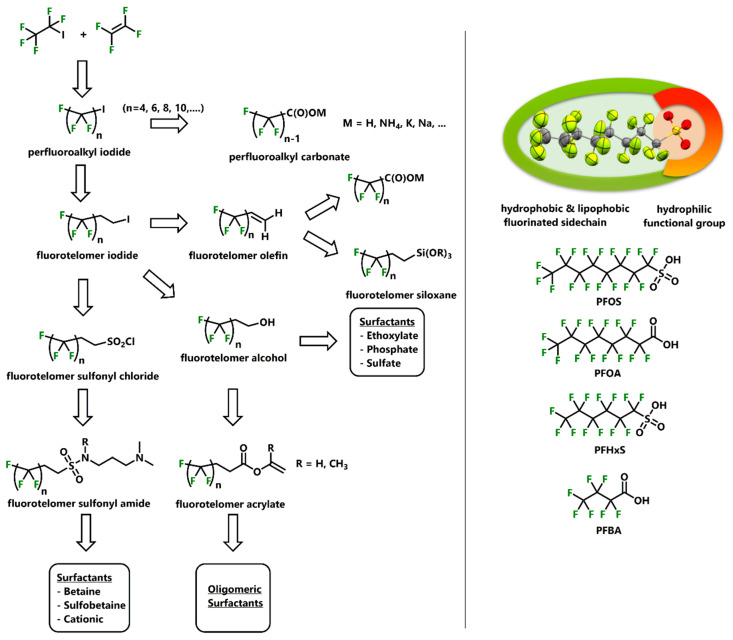
Left: schematic overview of synthetic production of per- and polyfluoroalkyl surfactants [6]. Right: highlighted amphoteric properties of linear perfluorinated surfactants; molecular structure of perfluorooctanesulfonic acid (PFOS), perfluorooctanoic acid (PFOA); perfluorohexanesulfonic acid (PFHxS) and perfluorobutanoic acid (PFBA).

Industrially, per- and polyfluorinated surfactants were synthesized mainly by electrochemical fluorination (ECF) or oxidation of either fluorotelomer alcohols (FTOHs) or fluorotelomer sulfonyl chlorides (FTSCls). Both classes can be generated either by hydrolysis or the reaction of Na_2_S_2_O_4_ and *N*-Chlorosuccinimide (NCS) with the respective fluorotelomer halogens (FTHals) [9]. In contrast to the previous mentioned methods, perfluorinated alkyl acids and carboxylates were mainly produced by the oxidation and hydrolysis of perfluoroalkyl iodides (Figure 1). Due to their polyfluorinated chain structure, PFASs exhibit extraordinary chemical stability and can be described as both strongly lipophobic as well as hydrophobic (Figure 1) [6,10,11]. Compared to hydrocarbon-based surfactants, fluorinated surfactants exhibit significantly stronger pK_a_ values due to their stronger electron-withdrawing substituents. Thus, trifluoro acetic acid (CF_3_COOH, pK_a_ = 0.52) exhibits a significantly stronger Brønstedt acidity, compared to the non-fluorinated analogue acetic acid (CH_3_COOH, pK_a_ = 4.76) [12]. Additionally, a higher degree of fluorination can be correlated to an increasing tendency towards formation of intra- and intermolecular interaction, mainly hydrogen bonding [13]. Moreover, most PFASs exhibit a much lower surface tension compared to their non-fluorinated derivatives, leading to unique wetting properties used in applications of, e.g., detergents [14]. Displaying a broad variety of unique properties, research and development intensively propelled the evolution of existing PFAS compounds over recent decades. Consistently, there are more than 4700 known and partly characterized compounds and precursors, respectively [15].

The enormous application potential of PFASs is contrasted by the lack of proper knowledge about the effects on flora and fauna and biocompatibility with respect to living beings, especially their harmful behavior towards human life. In 2001, the topic gained broader interest and attention after initial reports of the ubiquitous spread of PFASs and their impact on biological systems through environmental exposure [16]. Follow-up studies showed negative influences of PFASs on human metabolism and linked higher rates of physiologic diseases (e.g., lipid profile, diabetes or hypertension) [17,18]. In addition, several reports point out the possible interconnection of long-term PFOA and PFOS uptake and resulting organ damage or higher cancer rates, respectively [19,20]. The increasing number of scientific reports and proper investigations about the environmental impact and toxicity of PFASs eventually led to political debates and restriction on production, distribution and use of single PFASs in consumer products [10,21].

In Europe, the production and distribution of very persistent perfluorinated compounds (chain length C_8_-C_14_) are regulated by the REACH-enactment. Since 2009, the production and commercial application of perfluorooctanesulfonic acid (PFOS) has been heavily restricted, being classified as a “persistent organic pollutant” (POP) by the Stockholm Convention [22]. Analogously, the C_8_ chained perfluorooctanoic acid (PFOA) has been prohibited since 2020, leading to an adjustment of production and application towards shorter C_4_-C_7_-derivatives or substitution by polyfluorinated telomers [23].

After overcoming initial concerns, the worldwide research and investigation of (non)volatile poly- and perfluorinated alkylated compounds with respect to their environmental outcome and human exposure was strongly increased [21]. PFAS exposure led to the contamination of drinking water systems [24,25,26,27,28,29,30,31], river and lake sediments [32,33,34,35,36,37], soils [38,39,40], crops [41,42,43] and animals [44,45,46], as shown by numerous investigations. Moreover, PFASs were detected in deep ocean water samples [47], in the atmosphere [48] as well as in the Arctic environment [49]. Human PFAS intake is caused by the exposure of treated consumables but can be mainly traced back to the human food chain. Exhibiting a highly inert and persistent character, PFASs can be detected in various ecological systems all over the planet a long time after their exposure [26,27,46,50,51,52,53]. It was reported that PFASs can be mobilized in soils through the uptake of water, leading to the expansion of contamination in; e.g., agriculturally cultivated land [54,55]. The continuous release of contaminants induced by ground water flows and further water intake can cause a secondary contamination of natural water sources and adjacent watercourses, respectively [28].

On the one hand, the increasing numbers of studies reveal the true impact of PFAS exposure in the environment, but simultaneously show the enormous lack of knowledge and practical remediation applications. Current decontamination strategies of PFAS-burdened soils mainly consist of adsorption methods using PFAS adsorbents for fixation of PFAS pollutants in the ground. A second option is the utilization of a “pump and treat” process, cycling polluted soils through a washing plant leading to the concentration of the pollutants in the fine fraction. Only a subsequent, high-energy consuming pyrolysis process guarantees the total destruction of all fluorinated organic contaminants. Both approaches are cost-effective and not intended for the direct decomposition of all PFAS contaminants [56,57]. Hence, there is a great demand for innovative developments and chemical treatment technologies, dealing with new strategies of tackling the PFAS problem which are described in the following sections.

## 2. State-Of-The-Art Liquid Reactions

One of the biggest remediation challenges of PFAS contamination is the high persistence of perfluorinated molecules, originating from the extraordinary stable C−F bond ($D_{0}^{C-F}\;$= 485 kJ/mol) [12]. Breaking these bonds requires a significantly higher amount of energy than used for the chemical degradation of chlorinated ($D_{0}^{C-Cl}\;$= 323 kJ/mol) and brominated ($D_{0}^{C-Br}\;$= 269 kJ/mol) organic pollutants [58]. To lower the required energy amount, the use of catalytically active materials can be advisable [59,60,61]. The latest developments of advanced oxidation processes (AOPs), including chemical oxidation (e.g., persulfate oxidation, ozonation), photooxidation, electrochemical or thermal oxidation, have been recently reviewed by several authors [62,63,64,65]. In contrast to oxidative processes, reduction involves the direct transformation of electrons from a reducing agent with a lower reduction potential compared to the substrate. Advanced reduction processes (ARPs), reductive metals (e.g., zero-valent iron (ZVI)), hydride radicals (H·) and most notably hydrated electrons (E^0^ = −2.9 V) have been used to achieve the effective cleavage of C−F bonds in PFASs [66,67,68,69,70,71,72,73]. The investigation of PFAS reduction reactions have shown that they are sensitive towards the choice of reagents, their concentrations and reaction times as well as pH value of the solution [74,75,76], thus can show variable efficiencies. Hereafter, we give a detailed overview of recent developments of PFAS-degradation based on reductive defluorination.

### 2.1. Hydrothermal Treatment

The thermal decomposition of PFASs takes place between 200 and 800 °C [77]. In the absence of hydrogen sources, incomplete degradation takes place and usually yields shorter, more volatile PFASs and also exhibits a high global warming potential. For example, Sörengard et al. treated PFAS-contaminated soils directly in a furnace at 150–550 °C, where almost all added PFASs were degraded [78]. Additionally, the authors stated that supplementary air-phase vacuum filtration techniques are needed to separate the developing gaseous perfluoroalkyls. Yu et al. performed a hydrothermal liquefaction of wet PFAS-contaminated sewage sludge at 350 °C for 60 min [79]. More than 99% of perfluoroalkyl acids were degraded, but not totally defluorinated. In contrast, only a low amount of perfluoroalkyl sulfonates were degraded. In order to be converted into fuels, remaining short-chain PFASs require more sophisticated steps, including high temperature catalytic deoxygenation/denitration and cracking. Wu et al. used NaOH as additive for the hydrothermal degradation of PFOSs at 350 °C [80]. Subsequent ^19^F nuclear magnetic resonance (NMR) spectroscopy measurements showed a complete conversion of C−F bonds to fluoride (2.5 g/L PFOS) within 40 min for a reaction with 1 M NaOH. Small quantities of short-chain perfluoroalkyl acids (≤1.5% of inserted PFOSs) were detected as transient intermediates, indicating that an initial OH^−^-catalyzed cleavage of the sulfonate headgroup is followed by rapid sequential decarboxylation reactions, eventually leading to complete mineralization. Wang et al. recommended calcium hydroxide as the most effective Ca reagent for the PFAS defluorination of waste material at 400 °C because the carbon−fluorine bonds in PFASs can be converted to carbon−hydrogen bonds via the hydrodefluorination reaction [81]. Furthermore, PFASs with different chain lengths and functional groups were investigated showing that perfluoroalkyl sulfonates were more stable compared to perfluoroalkyl acids towards Ca treatment. 

### 2.2. Biochemical/Microbial Degradation

In general, degradation by microorganisms (biodegradation) is one of the most important mechanisms by which organic contaminants are removed from the environment. The reductive dechlorination of aromatic compounds has been demonstrated for chlorinated benzenes, phenols, benzoates, biphenyls, and dioxins by bacteria in sediments [82]. However, precise biodegradation pathways and the ultimate fate of PFASs are still unknown [82]. Additionally, the strength of the carbon–fluorine bond is generally believed to be the main factor in limiting the mineralization of perfluorinated molecules. Thus, most microbial studies on PFASs only show a very low degradation rate of <5% [83]. Often, only the carboxylic C−C bond is degraded leading to the formation of shorter chain PFASs [84]. Sun et al. showed that several fluorotelomer alcohols can be biologically degraded to trifluoro acetic acid (TFA), the shortest perfluoroalkyl acid, during 32 days in a landfill soil microbial culture system [85]. Furthermore, Luo et al. showed a degradation of PFOA by enzyme-catalyzed oxidative humification reactions with soybean meal as mediator [86]. In the presence of soybean meal and laccase, PFOA was degraded 24% in water after 36 days and 40% in soil slurry after 140 days. However, only C−C bond degradation could be monitored, yielding various partially fluorinated organic compounds.

### 2.3. Photochemical Reduction

Oxidative and photochemical methods were often investigated in several research projects in the past [62,76,87]. The photochemical reduction was mainly performed with UV light and free reducing radicals formed from sulphite (SO_3_^2-^), but other inorganic and organic compounds were also used. Song et al. successfully used UV light with sulphite for the defluorination of PFOA [67]. Their process led to a quantitative removal of PFOA after one hour and a defluorination ratio of 88.5% at reaction time of 24 h under an N_2_ atmosphere at 25 °C and a pH value of 10.3 in water. They also showed that NO_3_^−^ has a negative influence on the degradation of PFOA. Gu et al. used a similar system and degraded 98% of PFOS at 25 °C and a pH of 9.2 with a defluorination rate of approximately 50% after 30 min [88]. Bentel and co-workers operated the UV/sulphite system with a higher pH of 12 [75]. This approach led to defluorination rates of 73–100% for various perfluoroalkyl acids after an 8-h treatment. This finding was assigned to an elevated OH^−^ concentration in solution since the degradation of PFASs in the presence of pure NaOH was reported previously (see also Section 2.1). In a different report, various perfluoroalkyl ether carboxylic acids (PFECAs) were degraded at a pH of 9.5 [73]. All compounds were degraded but the defluorination rates were only between 30 and 98% after 48 h of treatment. In a different study, the PFECA GenX (perfluoro(2-methyl-3-oxahexanoate ammonium salt) was degraded and defluorinated in a UV/sulphite system at pH 10 [89]. This resulted in a defluorination rate of >90% after a six-hour radiation treatment. In a comparable experiment, these authors performed a degradation of the PFAS monochlorinated-polyfluorinated-ether sulfonate (F-53B) from the chromium industry with a defluorination rate of 70% after a two-hour treatment [69]. Tenorio et al. used an AFFF concentrate as a PFAS source for the UV/sulphite photo reductive treatment at 20 °C and a pH of 9.5 [90]. The experiment resulted in an overall PFAS defluorination rate of 53% after 49 h treatment.

Another approach was taken by Qu et al. [66]. They used potassium iodide as a mediator for the UV treatment. Their experiment with PFOA was conducted under anaerobic conditions at pH 9. The resulting defluorination rate of PFOA reached 98.9%. Tang et al. used a Fenton system (Fe^II^SO_4_/H_2_O_2_) for the photo reductive UV treatment of PFOA [91]. Within a reaction time of one hour, the added PFOA was rapidly degraded, resulting in a PFOA removal rate of 87.9% and a defluorination rate of 35.8%. After a reaction time of five hours the PFOA degradation rate and defluorination efficiency increased up to 95% and 53.2%, respectively. A photo reductive treatment with H_2_O_2_ and a water soluble tungstic heteropolyacid photocatalyst (H_3_PW_12_O_4_∙6H_2_O) was published by Hori et al. [92]. The combination of the photo active additives led to a defluorination rate of 88% after 24 h treatment. Gallium^III^ oxide was used in two studies for the photo reductive treatment of PFOA [93,94]. After three hours of UV treatment, 98.8% of PFOA was degraded and the defluorination rate was 31.6% under anaerobic conditions. Sahu et al. used a bismuth phosphate photocatalyst (Bi_3_O(OH)(PO_4_)_2_) for the UV treatment of PFOA [95]. This material showed quantitative PFOA degradation after one hour and a defluorination rate of approximately 60% after two hours, which was much higher than for other catalysts (Ga_2_O_3_, BiPO_4_ and TiO_2_) in this experiment. Additionally, TiO_2_ was utilized in combination with oxalic acid for the photocatalytic UV reduction in PFOA [96]. Under anaerobic conditions approximately 85% of PFOA was degraded and a defluorination rate of 16.5% was obtained after three hours of reaction time. Under aerobic conditions, no defluorination was possible. Furthermore, a combination of 3-indole-acetic-acid and an organo-modified montmorillonite was used [68]. In this reaction, the modified clay acts as electrostatic sorbent for PFAS molecules and simultaneously it can be used as stabilizing agent for hydrated electrons originating from photo excited 3-indole-acetic acid radical cations. For PFOA, quantitative degradation was obtained after five hours of treatment with a UV light accompanied by a defluorination rate larger than 90% within 10 h and up to 96% after 18 h treatment. 

### 2.4. Hetero- and Homogenous Redox Catalysis

Additionally, heterogenous and homogenous catalysis were also studied in different ways to degrade and defluorinate PFASs in aqueous media. Hori et al. treated PFOS combined with iron powder in aqueous solution which was heated at 150–350 °C under an elevated pressure of 23.3 MPa for six hours [97]. When temperatures above 250 °C were reached, PFOSs could be degraded. At 350 °C, the degradation of PFOSs was completed but only approximately 50% of the fluorine mass was mineralized. Two different studies used titanium^III^ citrate and vitamin B12 for the reduction of PFASs [98,99]. Exposed to anoxic aqueous conditions at 70 °C and pH 9, PFOSs were completely degraded to shorter chain derivatives by Ochoa-Herrera et al. Since reductive defluorination was the limitation in this approach, only 18% PFOS was defluorinated after seven days of reaction time [98]. Lee et al. used copper nanoparticles in addition to titanium^III^ citrate and vitamin B12, resulting in a 65% degradation of PFOA after 24 h in an anoxic aqueous solution at 70 °C and pH of 9 [99]. Liu et al. modified the procedure of Ochoa-Herrera et al. and used a cobalt catalyst to degrade PFASs [100]. Additionally, NiFe supported on activated coal was utilized as a heterogenous catalyst in order to degrade PFOS at 50 °C. Interestingly, 94% of PFOSs could be degraded and defluorinated after a long lasting 120 days period at 22 °C [101]. Another approach was presented by Liu et al. where ZVI and biochar were applied to reduce seven different PFASs in aqueous solutions [102]. Using the combination of both reagents was more efficient than the single application of ZVI because of the better adsorption capacity of biochar for PFASs. The PFAS removal rate was between 20 and approximately 95% after 120 days of reaction time, depending on the kind of PFAS. However, the defluorination rate for PFOA and PFOS could never exceed 10% and 5%, respectively, which might be underestimated by the sorption of fluoride by ZVI and biochar.

## 3. Mechanochemical Decomposition of PFASs and Involved Reaction Mechanisms

A far more promising strategy in terms of the decomposition of fluorinated pollutants in solid matrices might be the application of ball milling. Although mechanochemistry (MC) was intensively studied with regard to the removal of chlorinated and brominated persistent organic pollutants (POPs), little is known about the mechanochemical impact on PFASs [103,104,105]. Until today, only few examples have been reported showing the chemical degradation of representative compounds—mainly PFOS and PFOA—in ball-milling chemistry. Since this technique delivers high amounts of friction energy required to trigger the oxidative or reductive decomposition of perfluorinated substances, specific attention is drawn to several MC approaches in this review. Two main MC reaction types were classified by Dubinskaya—one including the mechanical stress causing the deformation of the crystal lattice and molecular structure, leading to the disruption of intermolecular interactions. Second, mechanical friction causes valence bond deformation, leading to the cleavage of chemical bonds, oxidation and hydrolysis reactions of organic molecules. Additionally, ball milling-induced friction generates free radicals on the substrate surface, leading to the formation of radical pairs and excited states [106,107,108]. These types of reaction are fundamental for MC reactivity and can be applied to the explanation of complex reaction pathways. Known MC processes for decomposition of PFASs are summarized and described in detail in the following sections. 

### 3.1. Lewis Base/Brönstedt Base-Assisted Reactions

A ground-breaking development was achieved when Baron and co-workers reported the mineralisation of polyvinylidene fluoride (PVDF) in the presence of dried sodium hydroxide to NaF via nucleophilic exchange after one hour of ball-milling treatment [109]. The latter was performed with a zirconia pot and balls operating at 700 revolutions per minute (rpm) under ambient conditions. X-ray diffraction (XRD) and ion chromatography was used to show the formation of mineralized NaF and consumption of the polymeric vinylidene fluoride. Since the formation of water was observed, the suggested mechanism proposed the formation of polyvinyl alcohols, leading to subsequent condensation reactions to branched polyethers or -ketones. Most notably, a 90% defluorination of PVDF could only be achieved by the consequent removal of water during the milling treatment. Similar results on the degradation of PVDF were reported during the same year with the use of another basic compound. Zhang et al. showed a 98% reduction in the same starting material, when ball milling was applied in the presence of La_2_O_3_ for at least 240 min under identical reaction conditions [110]. Again, the use of XRD and FT-IR spectroscopy could prove the gradual reduction in PVDF and the formation of LaOF as a stable fluoride-containing product. Mechanistically, the authors claim the exchange of two fluoride atoms by one O^2-^, leading to the formation of polyketones or polyvinyl alcohol which can undergo intermolecular condensation reactions [110]. Shortly after this, La_2_O_3_ was used in the mechanochemical defluorination of polytetrafluorethylene (PTFE) and was ground under the exact same reaction conditions. The reaction was reported to emanate from the oxide/fluoride exchange reaction leading to the formation of water, HF and CO_2_. Besides LaOF, La(CO_3_)F was observed as the primary mineralization product. When the reaction was performed at 600 °C, a decarboxylation reaction of the La(CO_3_)F was initiated, increasing the LaOF formation under the decomposition of PTFE [111]. In a similar manner, Zhang et al. performed the mechanochemical decomposition of PTFE with the aid of CaO and SrO, respectively [112]. The mineralization of organic fluorine to the very low soluble inorganic salts CaF_2_ and SrF_2_ as well as formation of CO gas, act as the driving force for breaking the strong C−F bonds of the fluorinated polymer. When SrO_2_ was used, the formation of SrF_2_ and direct carbonation of PTFE was observed. Analogous to previous experiments, the MC decomposition of polytetrafluoroethylene could be performed in the course of several hours.

**Figure 2:**
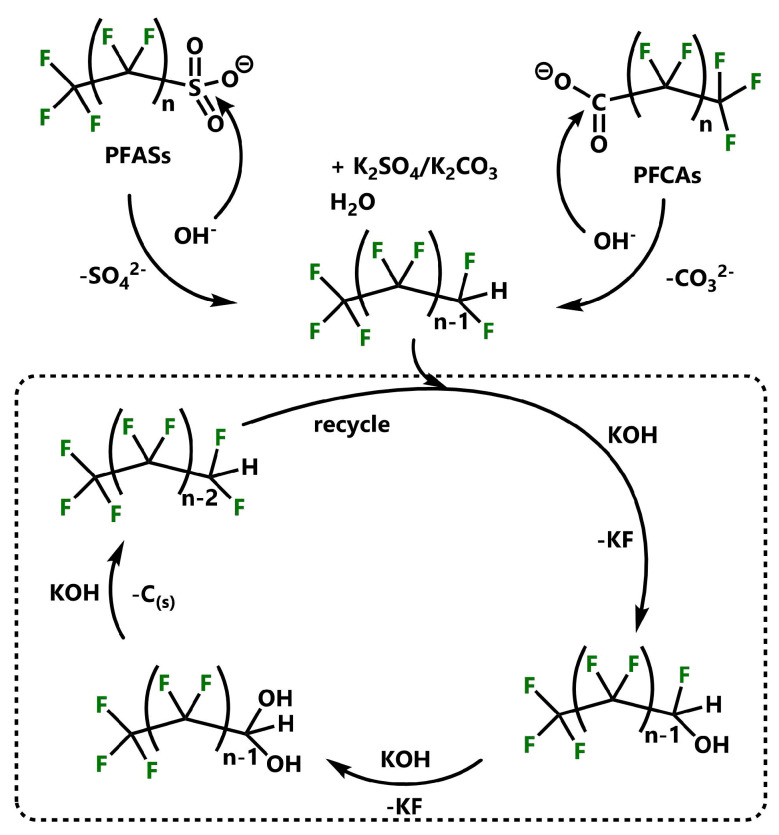
Proposed mechanism of reaction of KOH with PFOS and PFOA under mechanochemical treatment [114].

The first notable tribochemical experiments on perfluorinated surfactants were carried out by Hosomi et al. in 2008 [113]. The group was able to fully decompose two of the most abundant PFASs: perfluorooctanesulfonic acid (PFOS) and perfluorooctanoic acid (PFOA). The reactions were performed in steel containers loaded with a mixture of PFASs and CaO with a molar ratio of 2.0:2.1 (F:Ca). After an 18 h milling time at 700 rpm, no further decomposition of either PFOS or PFOA was detected. XRD diffractograms and FT-IR spectra were recorded to monitor the formation of CaF_2_ and CaCO_3_ as mineralization products. Although no overall yields were reported, the successful use of a fluoride-accepting metal oxide as co-milling agent for the decomposition of molecular PFASs was shown for the first time. In 2013, a detailed study on the mineralization of PFOS and PFOA was published with the use of a nucleophilic base KOH [114]. After 4 h of ball milling at 275 rpm, an 88.3 and 96.7% fluoride recovery from PFOSs and PFOA was measured, due to the higher stability of the PFOS molecule towards nucleophilic attacks. After 6 h, the respective XRD patterns showed a complete defluorination and formation of KF and K_2_SO_4_/K_2_CO_3_, respectively. Interestingly, the reaction could only be conducted when an excess of the reagent KOH was used. The mechanism for the KOH-induced defluorination reaction of PFOSs and PFOA was proposed via a primary nucleophilic attack of a hydroxyl anion at the carbon atom of the terminal carboxylic acid group (PFOA) or at the sulfur atom of the terminal sulfonate group (PFOS). After the cleaving of the carbonate/sulfate group, a second hydroxide anion can undergo a nucleophilic substitution of one terminal fluoride atom leading to the formation of KF and polyfluoro alcohols. The subsequent exchange of fluoride with hydroxide eventually leads to the formation of water and carbon as well as KF (see also Figure 2). Due to FT-IR spectra, a fast degradation of both starting materials could be monitored after only 30 min, hence the authors claim the initial steps to be the fastest, whereas after the decarboxylation and loss of SO_4_ the reaction rate slowed down gradually. 

Similar reaction rates were observed for homologous perfluorohexane sulfonate (PFHxS), perfluorobutane sulfonate (PFBS), perfluordecanoic acid (PFDA) and perfluorododecanoic acid (PFDoA), showing the high variety and low selectivity of the MC treatment [114].

**Figure 3:**
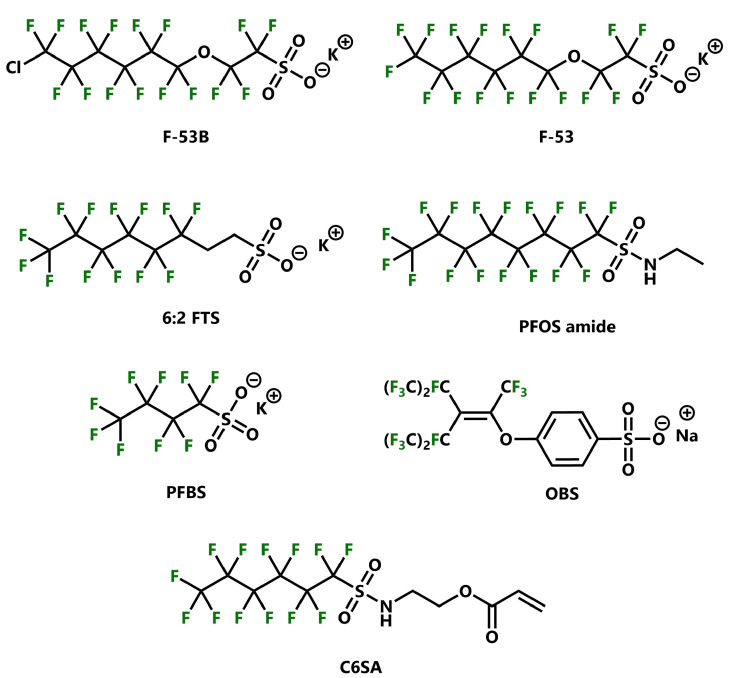
Overview of selected PFASs derivatives activated in tribochemical reactions. First row left: chemical structure of monosubstituted 2-[(6-chloro-1,1,2,2,3,3,4,4,5,5,6,6-dodecafluorohexyl)oxy]-1,1,2,2-tetrafluorethane sulfonate potassium salt know as F-53B. First row right: chemical structure of 2-[(1,1,2,2,3,3,4,4,5,5,6,6,6-tridecafluorohexyl)oxy]-1,1,2,2-tetrafluoroethane sulfonate potassium salt, abbreviated as F-53. Second row left: chemical structure of 1H,1H,2H,2H-perfluorooctanesulfonate potassium salt, short 6:2-FTS. Second row right: chemical structure of N-ethylperfluorooctane-1-sulfonamide (PFOS amide). Third row left: Structure of perfluorobutyl sulfonate potassium salt (PFBS). Third row right: chemical structure of *p*-perfluorous nonenoxybenzene sulfonate sodium salt (OBS). Bottom: chemical structure of 2-[2-(perfluorohexyl)]-sulfonylmethylaminoethyl acrylate, C6SA.

Among hundreds of polyfluorinated chemicals, the PFOS derivative F-53B (see Figure 3) attracted more attention than others when it was first reported to be highly exposed in especially Chinese environmental soils and aquatic systems [115]. Having been used for decades mainly in the galvanic industry, the monosubstituted polyfluoroether sulfate was eventually rated and classified as a POP in the Stockholm Convention in 2009. 

Initial MC defluorination attempts were performed by Huang et al. when they investigated the decomposition of F-53 and F-53B in the presence of KOH [116]. Using a steel-housed setup with rotation speeds of 275 rpm and an excess of KOH reagent, a full degradation of both compounds was achieved after six to eight hours of milling time. Since the degradation of F-53B follows first-order kinetics the monochloro-substituted compound could be degraded much faster than its perfluorinated derivative F-53. Using XRD, XPS as well as ^19^F NMR spectroscopy, they showed that F-53B was not only attacked by OH^−^ at the sulfonic group, but also the cleavage of the energetically weaker C−Cl bond occurred. Ion chromatography was implemented to show the mineralization products being fluorides, chlorides, sulfates as well as formates. Another group of widely used PFOS and PFOA alternatives are polyfluoro telomers. Although this compound class is regarded as less toxic and bioaccumulative, their representatives are still mainly persistent due to their slow-going and incomplete biodegradation [117]. An MC approach was first reported for the fluorotelomer 6:2-FTS (see Figure 3) in 2017, as complete decomposition was reached in less than one hour of tribochemical treatment with an excess of KOH. Analysis of the reaction pathway clarified that the presence of the significantly weaker C−H bonds (D_0_ = 417 kJ/mol) [12] in fluorotelomers leads to an efficient cleavage of the PFAS molecule and thus to faster decomposition times [118].

**Figure 4:**
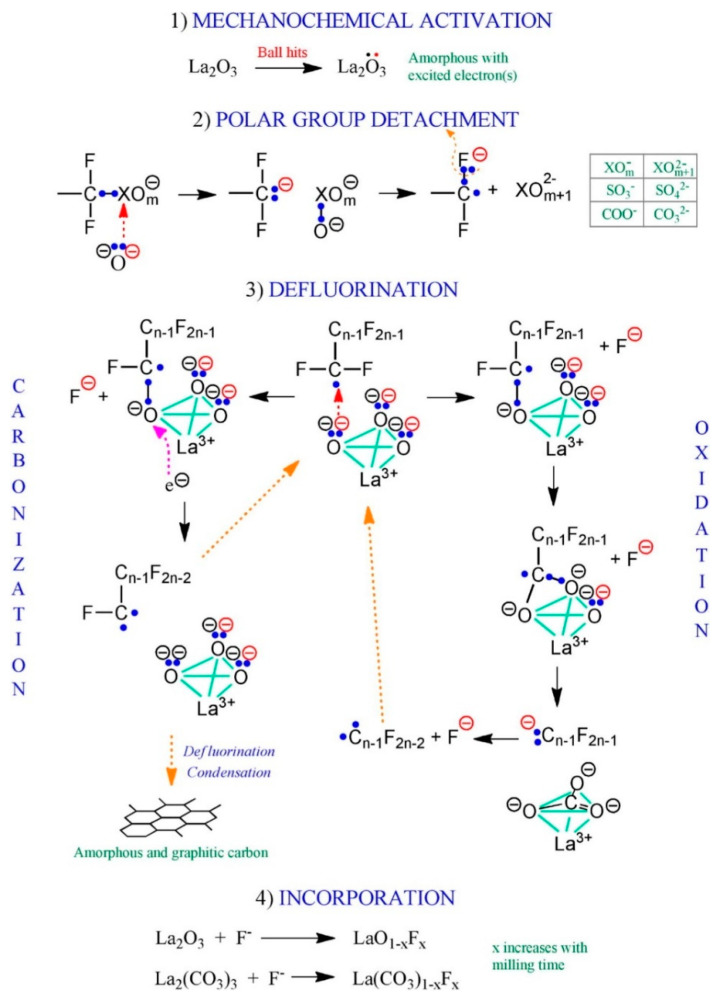
Proposed mechanistic pathways for the stoichiometrical activation of selected PFASs with La_2_O_3_ (reprint from [119], with permission from Elsevier).

While most scientific studies on MC defluorination reactions are based on the use of an excess of the respective co-milling agent, the first stoichiometric activation was contributed in the work of Huang et al. in 2017 [119]. In a series of experiments including fluorinated surfactants PFOS and PFOA as well as PFHxS, PFBS, OBS, PFOS amide and F-53B (see Figure 3), stoichiometric amounts of La_2_O_3_ were successfully applied to decompose all PFASs in a course of up to ten hours. The transformation of organic fluoride into the inorganic fluorides La(CO_3_)F and LaOF as well as the formation of LaSO_4_ could be observed via X-ray diffraction. Liquid chromatography coupled with tandem mass spectrometry (LC-MS/MS) studies as well as ^19^F NMR spectroscopy revealed no detectable residual organofluorine compounds, indicating a complete degradation and defluorination of the systems. Mechanistically, Huang et al. propose a multi-strategical approach (Figure 4) [119]. After the mechanochemically induced excitation of an electron, a nucleophilic attack of a negatively charged oxygen moiety (radical ion O^-.^) is proposed at the electrophilic carbon or sulfur atom. After the loss of CO_2_ or SO_3_, the defluorination process is launched by two possible mechanistic pathways. One pathway is the carbonization of the PFAS molecule, induced by the transfer of a surface charge electron from a La_2_O_3_ molecule on the PFAS residue, leading to the cleavage of a C−F bond and release of F^−^. Subsequent carbonization leads to a further release of fluoride ions and reduction in CF_2_ units to elemental carbon. The second proposed decomposition pathway of PFASs is induced by the oxidation of terminal carbon atoms by an oxidation agent such as La_2_O_3_. In this oxidative addition, the carbon is oxidized to R^F^-CO_2_ and reductively eliminated as La_2_(CO_3_)_3_, accompanied by the formation of La(CO_3_)F. Notably, the use of stoichiometric amounts of the Lewis base leads to a reaction rate of zeroth order, indicating its possible application as a catalytic system for PFAS degradation.

### 3.2. Lewis Acid-Assisted Reactions

**Figure 5:**
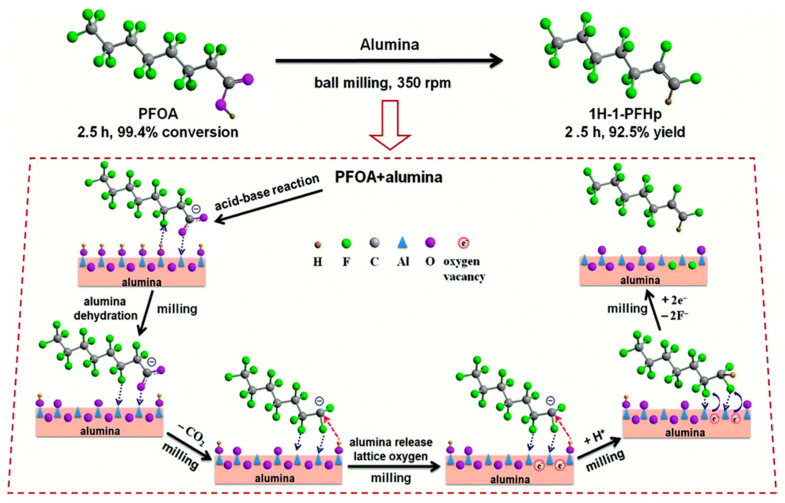
Proposed mechanistic steps for the alumina-mediated activation of PFOA (reproduced from [120] with permission from The Royal Society of Chemistry).

In 2018, Tang et al. reported an unprecedented use of an aluminum oxide in defluorination reactions, when they performed an MC reaction between PFOA and Al_2_O_3_, leading to a 99.4% removal and 92.5% conversion of the starting material to a polyfluoroalkene product after two and a half hours [120]. By implementing a different approach, PFOA transformation into a fluorinated building block was detected. Using an excess of Al_2_O_3_ (molar ration 1:25), an almost quantitative formation of 1*H*-perfluorohept-1-ene (1H-1-PFHp) was observed after two and a half hours of co-grinding in a planetary ball mill at 350 rpm. When the reaction was performed with the shorter derivatives, PFHxA and PFBA, quantitative conversion to the respective polyfluoroalkenes 1*H*-perfluorohex-1-ene and 1*H*-perfluorobut-1-ene was observed. The reaction products were identified via gas chromatography (GC)- and LC-MS and more specifically with the help of solid state and liquid NMR spectroscopy of the ^19^F and ^13^C nuclei. The formation of fluoride ions was traced by the application of a fluoride sensitive electrode (F-ISE). Bearing both Lewis acid aluminum sites and Brønstedt acidic OH functionalities at the surface, Al_2_O_3_ has been shown to be a promising candidate in the activation of organohalides [121]. Based on FT-IR spectra, the initial mechanistic step was proposed to be the deprotonation of the carboxylic acid group by surface hydroxyl groups of alumina (see Figure 5). After subsequent decarboxylation, the interaction of the negatively charged fluoride moieties and Lewis acidic aluminum centers on the surface leads to elimination of fluoride and reprotonation. Thus, formation of Al−F species as well as the respective polyfluoroalkenes occur. Analogous behavior has been studied during the MC activation of PTFE with Al_2_O_3_ [122]. Since the reaction barrier for a subsequent defluorination is too high, no further reductive dehalogenation reaction can be observed. With their approach, Tang and co-workers provided a method for the defluorination of a wide range of PFASs under sustainable and cost-efficient conditions.

Unprecedent work was recently published by Buryak et al., mechanochemically activating the fluoropolymer PTFE by various zero-valent metals in the presence of hexanes as co-solvents [123]. Using excess powdered Mg, Ti and W metals as co-milling agents, medium-scale batches of PTFE (300g mixture) could be mechanochemically degraded into gaseous fluorocarbon monomers. Alongside the organic by-products, water and CO_2_ were identified as the major reaction products. Although the fate of the reducing agent is not highlighted and the existence of fluoride ions were not traced, it is believed that zero-valent metals were reduced to the respective metal fluorides via reductive dehalogenation reaction induced by ball-milling electron transfer.

### 3.3. Oxidant-Assisted Reactions

Due to their extraordinary bond strength, the majority of PFASs are chemically inert towards chemical oxidation reactions. Nonetheless, several commercially available oxidants such as H_2_O_2_, O_3_ or KMnO_4_ have been shown to be reactive towards PFASs, based on free radical electron oxidation processes [124]. Although widely applied in advanced oxidation processes and solution-based remediation strategies of persistent organic pollutants, [64,125] sodium persulfate (Na_2_S_2_O_8_, PS) was rarely used in the MC decomposition reactions of PFASs. In 2015, the first noted report showed the MC activation of the fluoro surfactant derivative F-53B (Figure 3) by the co-milling of persulfate and NaOH in a planetary ball mill [126]. After eight hours of reaction time, the formation of H_2_O inhibited further reaction progress. Eventually, 88% of the precursor was decomposed while the fluoride recovery efficiency yielded over 50%. The authors point out that a specific molar ratio of PS and hydroxide is a key element for the effective generation of hydroxide, superoxide and sulfate radical anions, leading to the decomposition and defluorination of the reacted F-53B (PS:NaOH:F-53B = 4.17:1.75:0.05). The suggested mechanistic pathway involves the radical hydroxyl anions, initiating the cleavage of the C−S bond of F-53B and subsequent nucleophilic substitutions at the fluorinated organic intermediates leading to the gradual shortening and mineralization of the polyfluorinated chain. 

**Figure 6:**
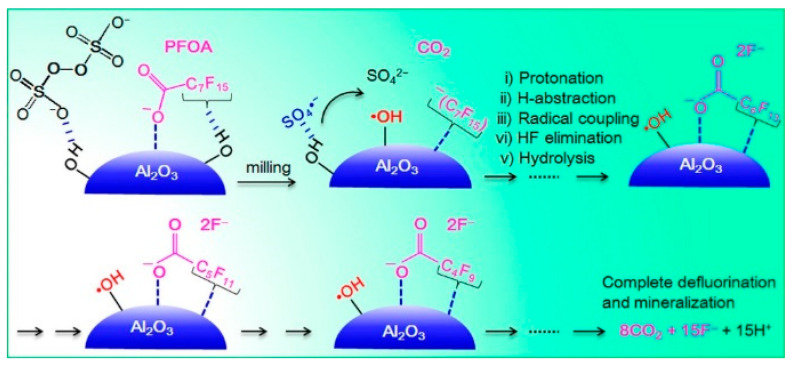
Proposed representation of synergetic influences of sodium persulfate (PS) and alumina during mechanochemical activation of PFOA (reprinted with permission from [127], copyright 2019 American Chemical Society).

By replacing the co-milling agent NaOH with alumina, the synergetic effects of Al_2_O_3_ and PS lead to the complete defluorination of PFOA under the formation of HF [127]. The co-workers alongside Zhu and Tang were able to almost quantitatively mineralize PFOA and its homologues perfluoropentanoic acid (PFPA), perfluorohexanoic acid (PFHxA) and perfluoroheptanoic acid (PFHpA) after two hours of treatment at 350 rpm in a planetary ball mill. Most notably, full defluorination could only be reached through the adjustment of the ideal co-milling agent to substrate ratio. As depicted in Figure 6, all PFAS derivatives were gradually consumed, resulting in the formation of CO_2_ and hydrogen fluoride. 

Mechanistically, an initial reaction step is the deprotonation of PFOA via the surface-bound oxygen atoms of Al_2_O_3_, whereas terminal hydroxyl functions act as catchers via intermolecular F···H−O interactions. High-energy ball-milling friction is claimed to cause the homolytic separation of PS, yielding sulfate radicals (SO_4_^−^·). The latter can undergo radical coupling reactions with surface hydroxyl groups to form strong hydroxyl radicals that are known to play a crucial role in the decomposition reactions of halogenated pollutants [76,124]. Isotopically labelled alumina (^18^O) was successfully implemented to prove the suggested reaction pathway and could partly be detected in the oxidized reaction products CO^18^O and C^18^O_2_, respectively. The reaction intermediates and products were analyzed and quantified via X-ray photoelectron spectroscopy (XPS), liquid ^19^F and ^27^Al NMR spectroscopy, HPLC MS/MS spectrometry and ion chromatography, respectively.

**Figure 7:**
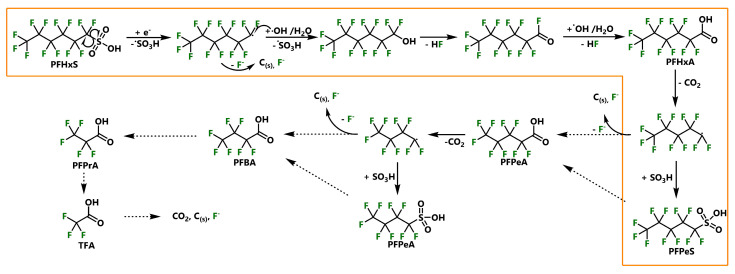
Decomposition of PFHxS via mechanochemical reaction with ferrate^VI^ and zero-valent iron (ZVI), proposed by [128].

Most recently, a unprecedented synthetic strategy was published, based on the complementary effects of zero-valent iron (ZVI) and the oxidation agent potassium ferrate^VI^ [128]. Complete degradation and almost quantitative defluorination (95%) was achieved when PFHxS (Figure 1) and the perfluoro methyl acrylate C6SA (Figure 3) were treated in a zirconia-housed planetary ball mill at 600 rpm for four hours. The optimized Fe^0^:Fe^VI^ ratio was found to be 2:1. Known as a potent reagent with a high reduction potential, ferrate^VI^ compounds have been successfully applied in various MC reactions [129,130]. ZVI has been used earlier in reductive defluorination reactions of PFASs before (compare Section 2.4) but serves a different purpose here. Upon the exothermic reaction of ferrate^VI^ with ZVI in the presence of water, H_2_O_2_ and Fe_2_O_3_ are formed (ΔG = −5913 kJ/mol). Simultaneously, ferrate^VI^ gets reduced by ZVI involving electron transfer reactions and the formation of perferryl^V^ and OH radicals. Both processes can be regarded as key elements in this tribo-assisted oxidation reaction. With regard to the mechanism of the decomposition reaction of PFHxS, the authors propose the homolysis of the relatively weak C−S bond as the favored initiating step, followed by the release of SO_3_H (see Figure 7). For methacrylate C6SA, an oxygenation reaction by one of the hypervalent iron oxide species leads to formation of an SO_3_ group at the fluorinated sidechain (not displayed). In the following step, the newly created fluoroalkyl radical can rearrange with an unpaired OH radical to form perfluoro alcohols. The elimination of HF leads to the formation of perfluoro acyl fluorides, which can be oxidized to perfluoro acids with a smaller chain length. Deprotonation and subsequent radical decarboxylation initiate the following reaction cycle, progressing the PFHxS decomposition. As shown by previously reported investigations, CO_2_, elemental carbon and HF were detected and quantified as the major reaction products. 

## 4. Future Prospect—Applicability in Remediation 

Surveying the existing literature concerning PFASs in environmental ambience of the last two decades, it is evident that reported cases of contamination have grown drastically, whereas new insights of PFAS degradation slowly progressed [48]. Both advanced oxidation and reduction defluorination processes (AOP, ARP) have been developed, utilizing different reaction conditions ranging from basic thermal treatments up to sophisticated photooxidation catalysts [62,76]. Their reaction efficiencies vary from low percentage yields to quantitative decomposition and full mineralization. While the majority of the reviewed processes seem both reliable and reproduceable, most of them require extreme reaction conditions. The latter range from high-demanding physical conditions (hydrothermal treatments) [78,80,131], over sensitive reactants and equipment (photochemical treatment) [62,67,90,91,132,133], up to very long reaction times (biological and bio-inorganic catalysis) [83,85,86]. 

In contrast, on a laboratory scale, the MC defluorination of PFASs is neither extremely energy demanding nor extremely sensitive, does not require extreme heat or pressure and reaction times stay within 1–18 h. Thus, mechanochemistry can be regarded as a pioneering technology in terms of PFAS remediation. Moreover, simple and cost-efficient reagents (CaO, ZVI, KOH, Al_2_O_3,_ La_2_O_3_, Na_2_FeO_4_, Na_2_S_2_O_8_, etc.) can be applied, yielding significant decomposition and defluorination ratios [113,118,119,120,127,128]. The process can be easily interrupted for quality control, process monitoring or addition of reagents or additives. Over the period of the last ten years, progress has been made with respect to the mechanistic understanding of tribochemical defluorination reactions. Consequently, reaction conditions and application of reagents (molar ratio, milling conditions, milling times, rotational speed) could be gradually improved. Nonetheless, further research on the isolation of single reaction products is necessary in order to get a profound mechanistic understanding of MC-controlled defluorination reactions. Due to its destructive nature, mechanochemistry allows specific reaction pathways that might not be accessible via other defluorination approaches. Furthermore, mechanochemistry does not require solvent assistance, hence is much more accessible for a wider range of applications. Altogether, mechanochemistry can undoubtedly be regarded as the most sustainable PFAS treatment technology existing.

So far, both reductive and oxidative defluorination reactions were performed under MC treatment. In comparison, more oxidative processes are known, since oxidation agents such as S_2_O_8_^2-^ are easily accessible and economically feasible. On the other hand, MC oxidative defluorination is more dangerous, since organically bound fluorine gets oxidized to HF, which is a highly toxic and corrosive gas. Only a subsequent base-wash converts HF into harmless mineral fluorides. By performing a reductive defluorination, no toxic HF gets formed since the direct conversion of organic fluorides into metal fluoride salts occur. This might be of greater interest when conceptions for scaling-up MC reactions are made. MC degradation has only been shown for a few selected PFASs on a small scale. With regard to availability as remediation tool, more detailed research is inevitable, especially in terms of the variety of fluorinated compounds, reagents and scalability. Furthermore, no solid information on matrix influences during MC defluorination reactions are available and limitations of milling systems, reaction conditions or efficiency of higher scale MC defluorination reactions are completely unknown.

Cagnetta et al. have summarized the previously attempted applications of ball milling as a means of remediation of POP-contaminated solids [105]. According to their investigation, large-scale ball mills performed their specific remediation tasks and competed well in terms of costs per m^3^ of contaminated soil (76 USD/m^3^), compared to state-of-the-art applications like incineration (914–1540 USD/m^3^) or landfilling (200–340 USD/m^3^). Nonetheless, reactions were performed on soils containing both organic chlorinated pollutants (PCB, etc.) and inorganic heavy metals (Cr, Pb, Ni, etc.) but no studies on ball milling PFAS-containing solid matrices are known. 

If up-scaled decomposition reactions might not work for PFAS-contaminated matrices, MC might contribute in a diverse way. In a different approach, the sorption capacity of chitosan was mechanochemically optimized and successfully applied as a sorbent for PFOS [134]. In the short term, improved PFAS adsorption techniques might be very beneficial since adsorption materials are often affordable and can be utilized quickly [57,65]. In the long term, PFAS sorption applications are not always reliable [135] and since the pollutant is only temporarily fixed, subsequent treatments are still inevitable.

## 5. Conclusions

In summary, we presented an overview of the state-of-the-art literature on reductive defluorination reactions on PFASs. Several different reaction strategies were introduced, and compared with regard to their reagents, reaction conditions, decomposition and defluorination efficiency. Most of the reported techniques only result in the C-C bond cleavage of PFASs, leading to formation of mostly poisonous short-chain fluorinated compounds. Furthermore, it must be mentioned that false treatment, e.g., thermal treatment, at too-low temperatures in the absence of a hydrogen source leads to an incomplete degradation, usually yielding shorter, more volatile PFASs. So far, complete PFAS decomposition is only enabled by thermal pyrolysis. Besides, the use of planetary ball mills has evolved over the last couple of years as a very promising and most importantly sustainable technology. Exhibiting an enormous reaction potential, mechanochemistry has developed as a competent application of decomposing per- and polyfluorinated POPs. A comprehensive understanding of the involved mechanistic processes can support further progression and improvements of reaction strategies. Since the limitations of existing methods have not been reached, further research and development is required to improve and to reach beyond the current state-of-the-art methods.

As stressed, there are no studies on implementing ball milling as a remediation application of PFAS-contaminated soils, ashes, waste or slags. So far, no scientific reports on the influence of solid matrices and the effects of reductive defluorination reactions during ball milling are known. More effort needs to be invested in optimizing reagents (e.g., appropriate reducing agents), milling conditions, influence of external parameters and milling efficiency. Additionally, the estimated cost calculation for MC degradation of PFASs is unknown but might be comparable to the estimated values of previous halogenated POP treatments. Thus, further testing is required to assure the functionality and reproducibility in larger scale applications.

Finally, the combination of several techniques such as the ball milling of PFAS-contaminated fine fractions concentrated in soil-washing plants or PFAS-loaded adsorption agents might be one example of a sustainable and effective future application of mechanochemistry as a PFAS remediation technology.
